# Characterization of lignocellulolytic activities from fungi isolated from the deep-sea sponge *Stelletta normani*

**DOI:** 10.1371/journal.pone.0173750

**Published:** 2017-03-24

**Authors:** Ramón Alberto Batista-García, Thomas Sutton, Stephen A. Jackson, Omar Eduardo Tovar-Herrera, Edgar Balcázar-López, María del Rayo Sánchez-Carbente, Ayixon Sánchez-Reyes, Alan D. W. Dobson, Jorge Luis Folch-Mallol

**Affiliations:** 1 Centro de Investigación en Dinámica Celular, Universidad Autónoma del Estado de Morelos, Cuernavaca, Morelos, Mexico; 2 Centro de Investigación en Biotecnología, Universidad Autónoma del Estado de Morelos, Cuernavaca, Morelos, Mexico; 3 School of Microbiology, University College Cork, Cork, Ireland; 4 Marine Biotechnology Centre, Environmental Research Institute, University College Cork, Cork, Ireland; 5 Instituto de Biotecnología, Facultad de Ciencias Biológicas, Universidad Autónoma de Nuevo León, San Nicolás de los Garza, Nuevo León, Mexico; Institute for Sustainable Plant Protection, C.N.R., ITALY

## Abstract

Extreme habitats have usually been regarded as a source of microorganisms that possess robust proteins that help enable them to survive in such harsh conditions. The deep sea can be considered an extreme habitat due to low temperatures (<5°C) and high pressure, however marine sponges survive in these habitats. While bacteria derived from deep-sea marine sponges have been studied, much less information is available on fungal biodiversity associated with these sponges. Following screening of fourteen fungi isolated from the deep-sea sponge *Stelletta normani* sampled at a depth of 751 metres, three halotolerant strains (TS2, TS11 and TS12) were identified which displayed high CMCase and xylanase activities. Molecular based taxonomic approaches identified these strains as *Cadophora* sp. TS2, *Emericellopsis* sp. TS11 and *Pseudogymnoascus* sp. TS 12. These three fungi displayed psychrotolerance and halotolerant growth on CMC and xylan as sole carbon sources, with optimal growth rates at 20°C. They produced CMCase and xylanase activities, which displayed optimal temperature and pH values of between 50–70°C and pH 5–8 respectively, together with good thermostability and halotolerance. In solid-state fermentations TS2, TS11 and TS12 produced CMCases, xylanases and peroxidase/phenol oxidases when grown on corn stover and wheat straw. This is the first time that CMCase, xylanase and peroxidase/phenol oxidase activities have been reported in these three fungal genera isolated from a marine sponge. Given the biochemical characteristics of these ligninolytic enzymes it is likely that they may prove useful in future biomass conversion strategies involving lignocellulosic materials.

## Introduction

Deep-sea organisms live under conditions that are likely to have resulted in the evolution of robust proteins over time in order to help them withstand adverse conditions such as high salinity, high pressures, and other abiotic factors [1–3]. Marine sponges (phylum *Porifera*) which are the most primitive pluricellular animals [4], often contain microbial communities that consist of symbiotic bacteria, archaea and unicellular eukaryotes. Microbial populations associated with different marine sponges from locations geographically distributed from around the world have been studied including the Great Barrier Reef [5], the Red Sea [6], the Mediterranean [7], the northern Atlantic [8], South America [9], China [10] and the Indo Pacific [11]. While bacterial populations derived from sponges have been extensively described, fungal communities associated with sponges remain relatively under studied.

Fungal diversity derived from a few marine sponges has been reported [12–15], in which orders *Eurotiales*, *Capnoidales*, *Pleosporales* and *Hypocreales* have been identified as commonly associated with various sponge species [11]. *Aspergillus* and *Penicilium* have also been reported as ubiquitous marine-derived fungi associated with sponges at different depths. Other genera which are frequently reported include: *Alternaria*, *Acremonium*, *Beauveria*, *Cladosporium*, *Curvularia*, *Eurotium*, *Fusarium*, *Gymnascella*, *Paecilomyces*, *Petriella*, *Pichia*, *Spicellum* and *Trichoderma* [11].

Fungal communities from the deep sea have to date been less well studied than terrestrial fungi and those colonizing superficial waters. Fungi associated with deep-sea sponges (500 to 1000 m in depth) are exposed to and are likely to have adapted to adverse/extreme environmental conditions present in the deep oceans (seawater below a depth of 200m), such as: low temperatures (<5°C), low levels of light, oligotrophic conditions, very high pressures (which increase by 105 Pascal (Pa) for each 10 m decrease in depth); together with oxygen-poor conditions and salinity levels of around 3.5% [16–19]. In the deep-cold habitats, the low temperatures place severe physicochemical constraints on the microbial growth, including a lower water activity (a_w_); accompanied with lower molecular diffusion, macromolecular interactions and metabolic reaction (enzyme kinetics) rates. The constraints of the stabilization of the secondary structure of DNA and RNA at low temperatures is also likely to negatively impact on transcription and translation; while other constraints include a decrease in water viscosity; a higher solubility of gases including oxygen, and consequently an increased risk from the production of reactive oxygen species [20–22].

Thus, to survive under these conditions they are likely to possess physiological adaptations facilitating cellular homeostasis in these deep cold environments. Most studies on marine-derived fungi associated with sponges, have focused on them as a potential novel source of bioactive metabolites for biotechnological applications such as anti-tumor, antibacterial, antiviral, toxin inhibitors, and anti-inflammatory metabolites amongst others [9,11,23] (For review see [24]). However, few studies describe the potential of sponge-derived fungi to produce lignocellulolytic enzymes.

The ability to degrade lignocellulose has been reported in over 30 phylogenetically diverse marine fungal strains [14,25,26]. Indeed marine-derived fungi are well recognized as a good source of enzymes of potential industrial interest (cellulases, xylanases, phenol oxidases, laccases, etc.); with strains exhibiting hydrolytic and oxidative activities being reported; for review see [27]. These fungal strains have predominantly been isolated from seawater, sediments, mangrove detritus and to a lesser extent from marine sponges [12,28,29].

The growing demand for new and more robust cellulases, xylanases and phenol oxidases for biotechnological applications (*i*.*e*., for biofuel production) has refocused biodiscovery efforts to novel environments such as the deep sea in an attempt to isolate and characterize new fungal strains with lignocellulolytic properties.

While cellulase production has been reported in marine-derived fungi such as *Chaetomium indicum* and mangroves isolates such as *Hypoxlon oceanicum*, *Julella avicenniae*, *Lignincola laevis*, *Savoryella lignicola* and *Trematosphaeria mangrovei* [30,31], there are limited examples of cellulases from fungi isolated from marine sponges. One such report involves members of the phyla Ascomycota and Basidomycotina isolated from *Haliclona simulans*, which produced cellulase activities [32]. Xylanases from marine-derived fungi isolated from soft corals [33], marine sediments [34] and from shallow water marine sponges in Antarctica [35] have been reported, with many possessing interesting biochemical characteristics (optimal pH, temperature and thermostability) with potential utility in biotechnological applications such as bioremediation and in the textiles and paper industries [27,35,36]. The phenol oxidase and esterase activities from sponge-derived fungi are however to date, greatly under studied.

Solid-state fermentation (SSF) is an efficient technology for microbial enzyme production and it is well established that fungi growing under SSF conditions results in the production of enzymes that are useful in the degradation of solid substrates such as bagasses [36,37]. SSF has many advantages over liquid fermentation such as: high volumetric productivity, relatively high concentration of microbial metabolites, and simple fermentation equipment and purification procedures [38]. SSF particularly offers several advantages in the utilization of agro-industrial residues which are under- or not-exploited [39]. Due to their ability to colonize a vast range of agro-based raw materials, fungi are microorganisms which are more extensively exploited in SSFs [38]. Particularly for lignocellulosic wastes degradation, solid fungal fermentation-based technologies have also been reported as a good strategy for agro-industrial residue degradation, and for the production of lignocellulolytic enzymes [40].

Although marine-derived fungi inhabit fully aqueous environments, they can successfully grow on different substrates under water-limited conditions. Many studies report the ability of marine-derived fungi to produce different metabolites when they are grown on agro-based residues under SSF conditions [41–43]. Additionally, many reports describe lignocellulolytic enzyme production by marine-derived and sponge-derived fungi growing on agro-based residues in the absence or near absence of free water [44,45]. Finally, there are also some reports demonstrating that the marine-derived fungus *Pestalitiopsis* sp. J63 is capable of enzyme production in SSF [46], with ligninolytic enzymes being produced [45]. Thus, SSF is an appropriate technology to investigate the potential of marine-derived fungi as a source of lignocellulolytic enzymes and other fungal metabolites. There are however, few reports to date describing the potential of sponge-derived fungi to produce lignocellulolytic enzymes on natural lignocellulosic materials.

With this in mind we focused on characterising the lignocellulolityc activity of fungi isolated from the deep sea sponge *Stelletta normani* which had previously been studied with respect to its resident prokaryotic microbiota [23]. Bacterial diversity has been described for a number of other *Stelletta* species including *S*. *kallitetilla* [47], *S*. *maori* [48], and *S*. *pudica* [47]; but there are no reports to date on fungi isolated from the genus *Stelletta* or from *S*. *normani* in particular. Given that *S*. *normani* had been collected at a depth of 751 m we reasoned that any fungi cultivated from the sponge are likely to possess interesting biochemical properties, enabling them to survive at the aforementioned extremes of temperature, light, salinity and pressure present at these depths [17].

In this work, we describe the isolation and characterization of three lignocellulolytic-halotolerant fungal strains from *S*. *normani*, which were identified as *Cadophora* sp. TS2, *Emericellopsis* sp. TS11 and *Pseudogymnoascus* sp. TS12. The strains displayed psychrotolerance and halotolerant growth on cellulose and xylan, with optimal growth rates at 20°C. They displayed halotolerant CMCase and xylanase activities at optimal temperature and pH values of 50–70°C and pH 5–8 respectively, while also successfully colonized maize stover and wheat straw. These strains also produced lignocellulolytic enzymes when they were grown on these natural lignocellulosic materials under SSF conditions. Given the biochemical characteristics of these enzymes it is likely that they may prove useful in future biomass conversion strategies involving lignocellulosic materials.

## Materials and methods

### Sponge sampling

Specific permission was not required to obtain the marine sponge samples used in this study, as they were collected in Irish territorial water, by an Irish research vessel, funded by the Irish government. The sponge samples do not involve endangered or protected sponge species. Sponge samples (*Stelletta normani*: Class *Demospongiae*, Order *Astrophorida*, Family *Ancorinidae*) were collected on June 2013, from a depth of 751 m from Irish waters in the North Atlantic Ocean (53.9861;-12.61) with the remotely operated vehicle (R.O.V) *Holland I* on board the *R*.*V*. *Explorer*. At the sampling location, the water temperature was 10.08°C with a pressure of 759.15 db and the density was 27.287 σ_T_ Kg/m3. The water salinity was 35.447 PSU and the conductivity was 3.894 S/m. The tissue samples were obtained *in situ* by excision of a piece (1–5 g) of one sponge and the species was identified by Bernard Picton (Ulster Museum) and Christine Morrow (Queens University Belfast). Upon retrieval, the sponge samples were washed with sterile artificial seawater (ASW) (33.3 g/L Instant Ocean, Aquatic Eco-Systems, Inc., Apopka, FL, USA) [23]. Samples were then placed in sterile plastic Ziploc bags and stored on dry ice for transport and subsequently used for fungal isolation.

### Fungal isolation

Ten grams of tissue belonging to a single sponge were macerated in 5 mL of sterile ASW, placed in a tube with sterile glass beads and vortexed. Primary isolation of fungi was performed by taking 1 mL of the macerated material with serial dilutions using sterile ASW being performed up to 10–5. One hundred μL of each dilution was inoculated on Petri plates containing either Malt extract agar-ASW or Potato dextrose agar-ASW (DIFCO) and cultures were incubated for 20 days at 20°C, as previously described [12]. Pure cultures of fungi were obtained from the primary isolation. Each colony from the primary isolation was picked onto a new Petri dish. Pure cultures were obtained after two passages, which were confirmed by microscopic/stereoscopy visualizations and morphological properties. The fungi were stored at 4°C in saline solution (0.5% NaCl) supplemented with glycerol (20%), and are conserved in the fungal collection of the School of Microbiology at University College Cork, under accession codes: TS2, TS11 and TS12.

### Taxonomic identification of fungal strains

Fungal mycelium from the strains TS2, TS11 and TS12 growing on Malt extract agar-ASW plates was collected following 10-days growth for genomic DNA isolation as previously described [49]. Four molecular markers (fragments of: 18S ribosomal DNA [50], 28S large sub-unit RNA gene (D1-D2 region) [51] and internal transcribed spacers 1 and 2 (ITS1 and ITS2 regions) [51]) previously described to be distinctive for the accurate molecular taxonomic identification of filamentous fungi were analysed. These gene fragments were amplified by PCR using the primers and conditions, as previously described [50,51].

Amplicons were analysed by electrophoresis (1% agarose gel) and purified using a commercial gel extraction kit (Thermo Catalogue No. K0513). Sanger sequencing was performed with the same primers used for the PCR reactions. The sequences obtained were deposited in National Centre for Biotechnology Information (NCBI) under accession numbers KR336667 to KR336677. The sequences were analysed by BlastN using the NCBI website (www.ncbi.nih.gov) and phylogenetic analysis was performed online with the server Phylogeny.fr (www.phylogeny.fr) [52,53].

From the different sequences retrieved from the BLAST hits, one of each species was used to construct the phylogenetic trees. In order to deduce the phylogeny of the fungal isolates ITS1, ITS2 and D1-D2 sequences were aligned from related fungi (the same Order or Family) held in culture collections [Agricultural Research Service Culture Collection (NRRL), American Type Culture Collection (ATCC), Canadian Collection of Fungal Cultures (CCFC), University of Alberta Microfungus Collection and Herbarium (UAMH) and Centraalbureau voor Schimmelcultures (CBS)] only where full length sequences were available. Alignments were concatenated using the Geneious software (version 7) [54].

The workflow setting in Phylogeny.fr included “*A la Carte*” mode step by step. MUSCLE was used for multiple alignments and they were curated in SeaView (version 4.6) [55]. The construction of the phylogenetic trees was based on two different methods and substitution models in order to improve the robustness of the final inferences. BioNJ (Neighbour-Joining) [56] and PhyML (Maximun Likelihood, ML) [57] programs combined with Kimura 2 Parameters (K2P) [58] and HKY85 [59] as substitution models were utilized, respectively. While the tree topology was bootstrapped 1000 times for BioNJ-based reconstructions, the branch supports were estimated under Approximate Likelihood Test (aLTR): Chi2-based parametric test for Maximun Likelihood-based phylogenies [60]. Finally the graphical visualization was performed by TreeDyn [61]. Ribosomal gene sequences from *Ustilago maydis* were used as an outgroup for tree rooting. All sequences considered in the phylogenetic analysis are listed in S1 Table.

### Growth rate determination

The specific growth rate of the three fungi (TS2, TS11 and TS12) (expressed as mm/day) was determined by inoculating plugs (7 mm in diameter) obtained from fungal pre-cultures grown in Vogel’s medium [62] supplemented with either 2% carboxymethycellulose (CMC) (Sigma) or 2% xylan (Sigma) in the same media and incubating them at different temperatures (4°C, 20°C, 30°C, and 40°C). The growth rate determination in saline conditions (NaCl) was determined by adding NaCl to the growth medium (0.5 M, 1.0 M, 1.5 M and 2.0 M, final concentration). Cultures were also grown without added NaCl. The diameter of the colony was measured every 24 h for 15 days. Experiments were performed in triplicate for subsequent statistical analysis of data.

### Fungal liquid cultures with CMC and xylan as sole carbon source at different NaCl concentrations. CMCase and xylanase activity determinations

CMCase and xylanase activities were measured both qualitatively and quantitatively. For qualitative determinations, 10-day cultures of the fungi (TS2, TS11 and TS12) grown on agar Vogel’s medium supplemented with 2% CMC or 2% xylan and NaCl (0 M, 0.5 M, 1.0 M, 1.5 M and 2.0 M, final concentration) at 30°C were flooded with 15 mL Congo red (1% *v/v* diluted in distilled water) for 15 min and washed 3 times with 20 mL of NaCl solution (1M). CMCase and xylanase activities were observed as discoloration halos around the fungal colonies [63]. Determinations were performed in triplicate.

For quantitative determination of both activities (CMCase and xylanase), 500 mL Erlenmeyer flasks with 100 mL Vogel′s medium (with 2% CMC or 2% xylan added as a carbon source) supplemented with NaCl (0 M, 0.5 M, 1.0 M, 1.5 M and 2.0 M, final concentration) were inoculated with plugs (7 mm in diameter) from pre-cultures of the fungi (TS2, TS11 and TS12) grown in the same solid media, and incubated for 10 days at 30°C and shaking at 150 rpm. Supernatants were recovered by centrifugation at 10,300 g for 10 min. Protein concentration was determined every 24 h using the Lowry method [64].

For both qualitative and quantitative cellulase determinations, we used carboxymethylcellulose sodium salt (Sigma, Product Number 360384) that contains 6.5–9.0 carboxymethyl groups per 10 anhydroglucose units and a sodium content of approximately 8% by weight, with 99.5% purity. Enzymatic activity was subsequently recorded as CMCase activity.

Enzymatic activities were also calculated every 24 h employing the 3,5-dinitrosalysilic acid (DNS) assay [65] and were expressed as IU/mg protein. The analytical procedures and volumes employed in the reaction mixture to determine both activities were as previously described [66]. Briefly, for CMCase and xylanases activity measurements, CMC and oat xylan (both 2%) dissolved in citrate buffer (50 mM and pH 5) were used as substrates. In each case, the reaction mixture contained supernatant from the liquid media (200 μL), 50 mM citrate buffer pH 5 (300 μL) and substrate solution (500 μL). The reaction was incubated at 30°C (in an attempt to identify robust enzymes capable to operating at ≥ 30°C) for 30 min and was monitored every 5 min. In summary, 50 μL aliquots were taken, mixed with DNS solution (50 μL), boiled for 5 min and then cooled on ice. The absorbance was measured at λ 540 nm in a spectrophotometer (BioMate, ThermoSpectronic). Glucose or xylose standard curves (ranging from 0.1 mg/mL to 2.0 mg/mL) were used to extrapolate the reducing sugar concentrations and the slopes were calculated to determine the velocity of the reaction. The concentration of released reducing sugars *vs*. time was used to calculate enzymatic activities. One international unit (IU) was defined as 1 μmol of glucose or xylose equivalent released per minute, under the assay conditions. Triplicate independent assays were performed and three readings for each sample were taken in all cases.

The supernatants recovered from these liquid cultures were used to study the influence of the temperature and pH on CMCase and xylanase activities, and both, thermostability and halotolerance.

### Optimal temperature and pH of CMCase and xylanase activities

Enzymatic reactions were performed as described earlier at different incubation temperatures (1°C, 10°C, 20°C, 30°C, 40°C, 50°C, 60°C, 70°C and 80°C) in 50 mM sodium citrate buffer, pH 5. Different pH conditions ranging from 2 to 10 were tested at the optimal temperature in each case in citrate (2 to 6) or phosphate (7 to 10) buffer depending on the pH being tested. All measurements were determined in triplicate.

### Thermal-stability of the CMCase and xylanase activities

An aliquot (500 μL) of each supernatant from liquid cultures was incubated at 30°C, 40°C, 50°C, 60°C, 70°C and 80°C for 1 h. Subsequently, supernatants were cooled on ice for 5 min. Following this, the enzymatic activities from the heat treated supernatants, were determined using the optimal conditions (temperature and pH) for each activity obtained as described in the previous section. The residual activities expressed in percentages were reported. All measurements were performed in triplicate.

### CMCase and xylanase activities in the presence of different salt concentrations

CMCase and xylanase activities in the presence of different salt concentrations were determined as previously described (employing the optimal temperature and pH for each enzyme) with the addition of NaCl to a final concentration of 0.5 M, 1.0 M, 2.0 M and 3.0 M in the reaction mixture. All measurements were performed in triplicate.

### Solid State Fermentation (SSF) on maize stover and wheat straw

SSF were performed using the following autoclaved substrates: maize stover (*Zea mays*) and wheat straw (*Triticum aestivum*). These substrates were selected because large quantities are produced worldwide and they have also previously been shown to be very useful for lignocellulolytic enzymes production [66]. Erlenmeyer flasks of 500 mL including 5 g of each substrate were inoculated with two plugs (7 mm in diameter) of each fungal strain (TS2, TS11 and TS12); which had previously been grown on Malt extract agar-ASW plates. Humidity in the system was maintained by adding 2.5 mL Vogel′s solution to the solid substrates. Fermentation was allowed to take place at 30°C for 12 days. Subsequently, the cultures (substrates with fungal growth) were collected, washed with 5 mL (50 mM citrate buffer pH 6) and the soluble fermentation products were recovered by filtration used Whatman^®^ filter paper and centrifugation at 10,300 g for 20 min.

The soluble products recovered from the SSF were used to determine CMCase, xylanase, peroxidase and phenol oxidase activities. Also, zymograms for CMCases and xylanases were performed.

### Enzymatic activities from SSF

CMCase and xylanase activities were determined as mentioned before (see CMCase and xylanase activity determinations section). Avicel (2%) was used as substrate to confirm avicelase activity, and the enzymatic reactions were performed under the same conditions described in section 2.5.

Peroxidase and phenol oxidase activities were tested according to the previously reported method using 2,2'-azino-bis(3-ethylbenzothiazoline-6-sulphonic acid (ABTS) as substrate for both complexes [67,68]. The ABTS concentration in the mixture was 2 mM and assays were performed in a 300 μL final volume. For phenol oxidase activity, reactions containing supernatants (50 μL) from fungal cultures grown under solid-state fermentation, ABTS (10 μL), and 50 mM acetate buffer pH 5 (240 μL) were used. Hydrogen peroxide (0.3%) was used for peroxidase determinations. In this case, 50 μL of supernatant, 10 μL of ABTS, 237 μL of acetate buffer and 3 μL of H_2_O_2_ were employed in the reactions. Enzyme assays were performed following published microplate (96 well) protocols [67,69]. Each reaction was incubated at room temperature for 5 min and then, the oxidation rate of ABTS to ABTS+ released was measured at 436 nm [67]. The ABTS molar extinction coefficient used was 73,000 mM-1 cm-1 and the calculations were performed as previously described [67,68]. The volumetric activities were obtained in IU defined as μmoles of ABTS+· formed from ABTS min− 1 (U) per mL− 1, and expressed as specific activities (IU/mg protein) considering the protein concentration in each sample.

Triplicate independent assays were performed and three readings for each sample were taken in all cases.

### Zymograms

Zymograms were performed to identify CMCases and xylanase isoforms from soluble products recovered from the SSF from the three strains (TS2, TS11 and TS12). Soluble products from the fermentations were obtained as previously mentioned (see SSF section). Zymograms were performed as described in Batista-García et al. [66] and Quiroz-Castañeda et al. [70]. Briefly, 20 μg of protein (without 2-mercaptoethanol and prior boiling) were loaded per lane in 10% polyacrylamide gel. Gels did not contain SDS, but SDS was added at 0.05% to the running buffer and after the run the gels were washed three times (40 min each) in PCA buffer (50 mM KH_2_PO_4_, 50 mM citric acid pH 5.2) in order to remove SDS. For CMCases, 2% CMC was added and polymerized with the gel, while 2% oat xylan was used in the gel for xylanases. Once the electrophoresis was carried out, gels were washed to remove the SDS and then incubated with a 1% Congo red solution (in water) for 30 min at room temperature and then washed 3 times with a 1 M NaCl solution. The CMCase and xylanase activities developed as clear bands, as the substrate is degraded resulting in loss of dye binding. The molecular weight of the bands was estimated against a protein marker (Fermentas Catalogue No. 26612).

### Release of fermentable sugars from cotton fibres

The saccharification potential of the fungi was evaluated, with each strain being grown in Vogel′s medium supplemented with CMC or xylan, and the supernatants recovered by centrifugation at 10,300 g for 10 min. Briefly, 5 mg of cotton fibres (pharmaceutical-grade) was treated with 25% NaOH for 15 min at 4°C, and subsequently washed five times with sterile distilled water to remove the alkali [71]. The same amount of protein (20 μg in total) from different cultures was incubated with the cotton fibres at the optimal temperature and pH in each case: *(i)* supernatants from fungal cultures grown in Vogel′s medium supplemented with 2% CMC, *(ii)* supernatants from fungal cultures grown in Vogel′s medium supplemented 2% xylan and *(iii)* mix of proteins (1:1) from both of the previous cultures *(i)* and *(ii)*. Additionally a negative control treatment was performed (cotton fibres incubated with phosphate buffer 0.1 M pH 5), and aliquots of 50 μL were taken for 8 h (sampling each every hour) following treatment. The release of fermentable sugars was determined using the DNS method as previously described [65].

Additionally, experiments were conducted to confirm the presence of cellulases (mainly exo-1,4-β-glucanases, since endoglucanases can not hydrolyse crystalline cellulose), in the supernatants from cultures of fungi grown on CMC and Xylan as carbon sources. For this purpose Avicel was used as a substrate for the reaction.

### Statistical calculations

For statistical treatment of experimental data, the arithmetic mean and the standard deviations were calculated. Simple classification ANOVA (variance analysis) tests were applied to determine significant differences between the different cases. Firstly, the assumptions of ANOVA were revised: analysis of homogeneity of variance (Hartley-Cochran-Bartlett test) and normal distribution (Kolmogorov-Smirnov and Lilliefors tests) were performed. Subsequently ANOVAs were conducted to demonstrate the similarities or differences between the data of the population of samples. Finally, a post hoc analysis that defines the order of the differences found in the ANOVAs was developed. The Fisher LSD, Tukey HSD and Duncan tests were performed for the post hoc analyses. The use of these three tests ensures greater statistical robustness of the proposed analysis. All statistical calculations were performed in Statistica v12.6 (https://support.software.dell.com/statistica/download-new-releases).

## Results and discussion

### Isolation and identification of fungal strains

Fourteen different strains were isolated from the *Stelletta normani* sponge samples. In all dilutions tested (serial dilutions up to 10–5), TS2, TS11 and TS12 isolates were the most abundant and preliminary screening for CMCase and xylanase activities showed them to be the best producers, as judged by the diameter and clarity of the halo/zones they produced. Genomic DNA was isolated from the strains to amplify and sequence the molecular markers ITS1, ITS2 and D1-D2 regions (from 28S ribosomal subunit) and 18S rDNA from each of the fungal strains. This allowed us to taxonomically identify each of the three fungal strains. The first hits for each new isolate and for the four ribosomal markers retrieved from blastn are found in S2 Table.

**Fig 1:**
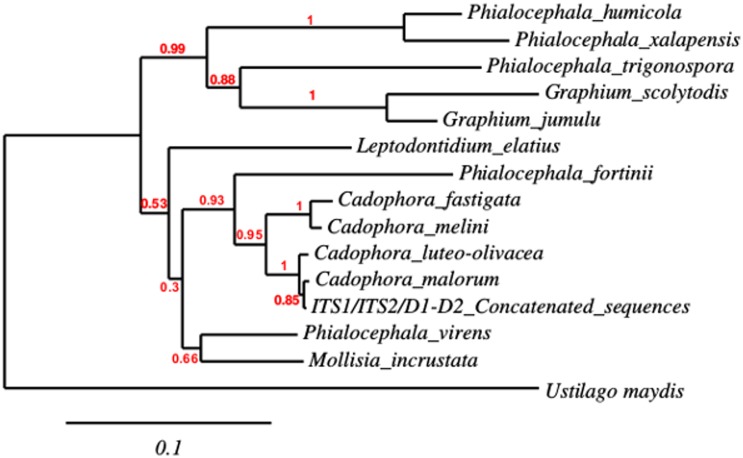
Phylogenetic reconstruction for the strain TS2. ITS1/ITS2/D1-D2 concatenated sequence-based phylogeny was constructed using NJ method with K2P as substitution model. Bootstrap values are indicated with corresponding nodes. Bar indicates the nucleotide substitution per site. *Ustilago maydis* was used as the outgroup. D1-D2, ITS2 and ITS1 sequences were deposited in the NCBI under accession numbers KR336670, KR336675 and KR336672 respectively. Accession numbers of the sequences used in this analysis are listed in S1 Table.

TS2′s ITS1/ITS2/D1-D2 concatenated sequence-based phylogeny was obtained by BioNJ using K2P as a substitution model, both of which have been previously used to estimate fungal phylogenetic relationships from ribosomal markers [72,73]. K2P has also been applied to study taxonomic divergence in different taxa such as bacteria and protozoa [74,75]. The phylogenetic analysis allowed us to propose that the TS2 isolate belongs to the *Cadophora* genus, with TS2 grouping directly with other *Cadophora* species (Fig 1). ITS1/ITS2/D1-D2 concatenated sequence-based phylogeny suggests that the most phylogenetically related species to TS2 is *Cadophora malorum* with a support value of 0.84 (Fig 1). TS2 is clustered in a clade exclusively including *Cadophora* species, which is supported with high bootstrap values for each branch. In general, the bootstrap values for this phylogeny (Fig 1) indicates the robustness of the tree. In addition, a phylogeny analysis using 18S rRNA also showed that TS2 is closely related to the *Cadophora* genus (S1 Fig).

*Cadophora* spp. have been isolated from diverse regions globally (United States, South Africa, Uruguay, Spain, Sweden and Canada) [76]. They have also previously been reported in Antarctica, where extreme weather conditions including UV radiation and high salt concentrations are thought to influence their growth [77,78]. To the best of our knowledge, this is the first time that a *Cadophora* species has been isolated from a marine sponge, which may help provide further insights into the distribution and various ecological niches of this fungal genus.

**Fig 2:**
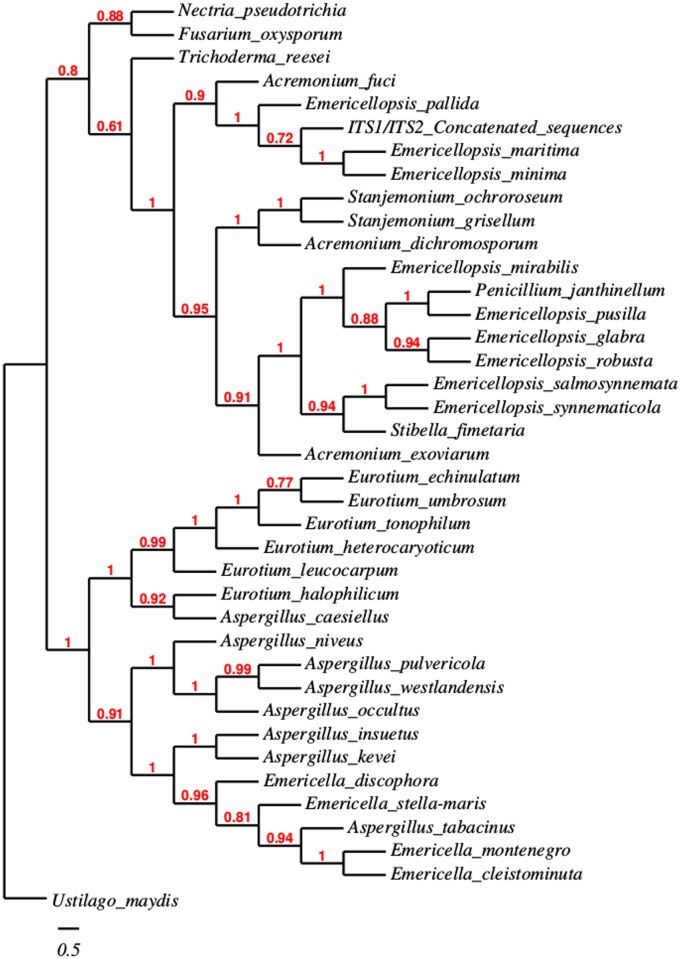
Phylogenetic reconstruction for the strain TS11. ITS1/ITS2 concatenated sequence-based phylogeny was constructed using ML method with HKY85 as substitution model. aLTR values are indicated with corresponding nodes. Bar indicates the nucleotide substitution per site. *Ustilago maydis* was used as the outgroup. ITS1 and ITS2 sequences were deposited in the NCBI under accession numbers KR336673 and KR336676 respectively. Accession numbers of the sequences used in this analysis are listed in S1 Table.

Regarding the TS11 identification, the phylogenetic analysis suggests that this strain belongs to the *Emericellopsis* genus with the phylogenetic tree employing a ITS1/ITS2/D1-D2 concatenated sequence (Fig 2), being the closest related species to *Emericellopsis maritima* and *Emericellopsis pallida* (Fig 2). The TS11′s concatenated sequence is placed in an independent branch between *Emericellopsis pallida* and a node grouping both, *E*. *maritima* and *E*. *minima* with robust branch support values (aLTR) of 0.82 to 1. The above analysis was performed using the ML method under HKY85 substitution model because in this case, acceptable bootstraps quality-values were not obtained when NJ/K2P was used for tree construction (data not shown). However, TS11′s concatenated sequence still clustered with the *Emericellopsis* genera, making our results fully congruent with a previous study based on rRNA genes that using different substitution models found a similar taxonomic relationship [79]. Consequently, ML and HKY85 as a substitution matrix were utilized for a robust phylogenetic inference. The phylogenetic relationship estimation using the ML method and HKY85 matrix system is also a good strategy for robust and high-quality tree constructions based on ribosomal genes [79]. Having said that, phylogenies obtained using ML/HKY85 have also been employed to establish taxonomic inferences in bacteria [80], fungi [81,82] and plants [83]. Finally, a phylogeny based on 18S rRNA gene was constructed and demonstrated that TS11 is related with *Emericellopsis* spp. (S2 Fig).

Although *Emericellopsis* spp. have been reported to share high homology with *Acremonium* spp. when comparing ITS and β-tubulin genes [84], but by using both the ITS1 and the ITS2 primers here, we were able to determine that TS11 belongs to the *Emericellopsis* genus (Fig 2).

*Emericellopsis* species have previously been isolated from a number of locations throughout the world and from several sources (skin from reptiles, agricultural and forest soils, peat, rhizomes, prairies, freshwater, estuarine and marine-mud sediments) with isolates from terrestrial and marine origins commonly forming different clades [84,85]. Moreover *Emericellopsis* spp. have also been isolated from different marine macroalgae and sponges and have previously been studied due to their ability to produce non-ribosomal peptide antibiotics [11,86–90]. They have also recently been reported to influence the community structure in photosynthetic microbial mats, by degrading the top photoautotrophic layer of these intertidal hypersaline mats [91].

**Fig 3:**
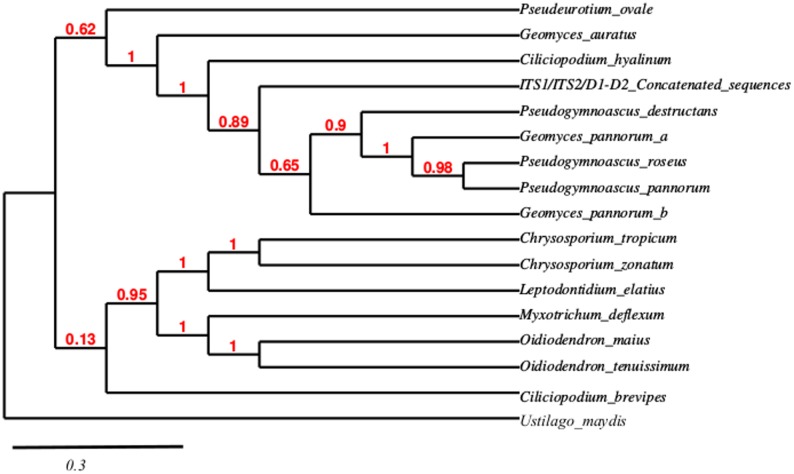
Phylogenetic reconstruction for the strain TS12. ITS1/ITS2/D1-D2 concatenated sequence-based phylogeny was constructed using ML method with HKY85 as substitution model. aLTR values are indicated with corresponding nodes. Bar indicates the nucleotide substitution per site. *Ustilago maydis* was used as the outgroup. D1-D2, ITS1 and ITS2 sequences were deposited in the NCBI under accession numbers KR336671, KR336674 and KR336677 respectively. Accession numbers of the sequences used in this analysis are listed in S1 Table.

Regarding strain TS12, the phylogenetic analysis based on ITS1/ITS2/D1-D2 concatenated sequence revealed that it belongs to the poorly defined *Pseudogymnoascus* genus (anamorph representatives of the *Geomyces* genus) (Fig 3 and S3 Fig), with *Pseudogymnoascus destructans* being the closest related species with a 0.89 as branch support value. TS12 is placed in a clade conformed by *Geomyces pannorum*, *Pseudogymnoascus roseus* and, *P*. *pannorum* (Fig 3). As in the previous case, this phylogeny was conducted by ML/HKY85, which evidences acceptable aLTR values indicating the robustness of the phylogenetic reconstruction. When this phylogeny was constructed by NJ/K2P the branch supports did not produce extensively high-quality values, however TS12′s concatenated sequence still grouped with *Pseudogymnoascus* species. Additionally, a phylogeny based on 18S rRNA gene was performed and revealed TS12 relationships with *Pseudogymnoascus* spp. and *Geomyces* spp. (S3 Fig).

*Pseudogymnoascus* spp. have previously been isolated from Antarctica with *Pseudogymnoascus* sp. being isolated from Antarctic soils [92] and *Pseudogymnoascus* sp. F09-T18-1 which has been shown to produce novel nitroasterric acid derivatives being isolated from the Antarctic sponge *Hymeniacidon* sp. [93].

A number of molecular markers have previously successfully been used for the taxonomic identification of fungal genera and species, including: the 18S rDNA gene [50], the mitochondrial cytochrome *b* gene [94], a putative toxin pathway regulatory gene (i.e. *aflR*) [95], the DNA topoisomerase II gene [96], the β-tubulin gene [97], the ITS regions between the small- and large-subunit rDNA genes [98], and the variable regions D1-D2 from 28S rDNA [51,98]. In particular the D1-D2 region from 28S rDNA and both, ITS1 and ITS2 have previously been successfully employed for fungal taxonomy purposes [51,66,99], as was the case here where they allowed the identification of our three fungal strains TS2 (*Cadophora*), TS11 (*Emericellopsis*) and TS12 (*Pseudogymnoascus*) to the genus level.

The role of these fungi in the deep sea sponge *S*. *normani* is not clear, but given that sponges are filter feeders, and that then they are likely to be exposed to pollutants that may be present in the seawater, and may accumulate impurities from phytoplankton, or other suspended matter; it is possible, that some of the sponge associated fungi may produce degradative/hydrolytic enzymes to acquire nutrients from these materials [100]. In addition as previously mentioned, marine derived fungi are believed to play an important role in detritus processing and in lignocellulose degradation, so these fungi may also play a role in ligninolytic processes [12,28,29]. Indeed a number of marine-derived fungi such as *Aspergillus sclerotiorum*, *Cladosporium cladosporioides* and *Mucor racemosus*, isolated from cnidarian samples in Sao Paulo Brazil, have been shown to produce high levels of lignin peroxidase, manganese peroxidase and laccase activity; all of which are important in lignin degradation by fungi [28]. While *Marasmiellus* sp. and *Tinctoporellus* sp. isolated from the Brazilian sponges *Amphimedon viridis* and *Dragmacidon reticulata* have also been reported to be good producers of laccases [29]. With this in mind we focused our attention on the ligninolytic ability of the three fungal strains *Cadophora*, *Emericellopsis* and *Pseudogymnoascus*, particularly given that ligninolytic enzymes from marine-derived fungi are likely to find utility in biotechnological applications with alkaline pH, high salinity, low temperature, oligotrophic conditions, low water potential and hydrostatic pressure, such as amongst others- the treatment of coloured industrial effluent and bioremediation in high salt concentration environments, biofuel production, deinking, laundry and in the food industry [11,27,101].

### Growth rate determination

**Fig 4:**
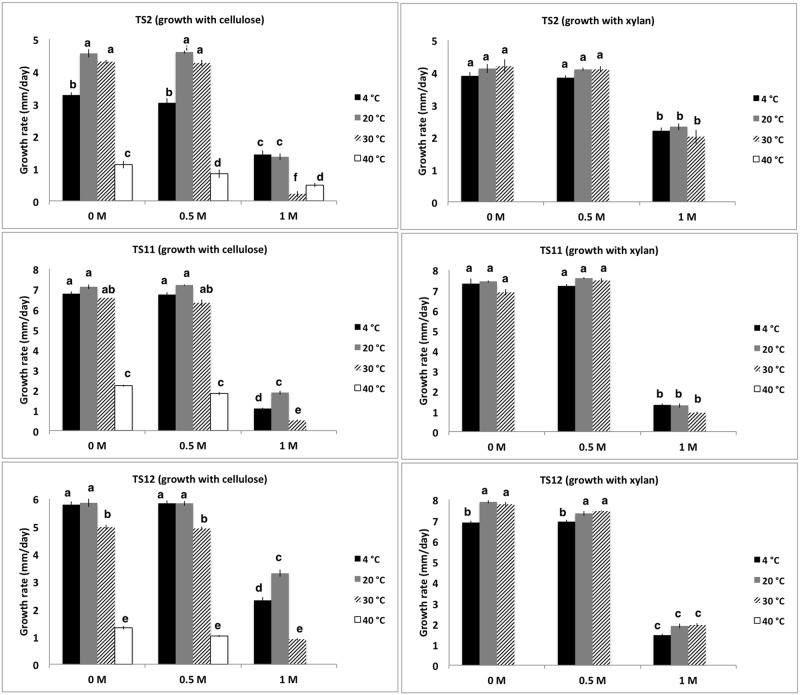
Growth rates for TS2, TS11 and TS12 at different temperatures and NaCl concentrations. Different letters (as superscripts) reflect statistically significant differences derived following ANOVA analysis.

The growth rate of the three fungal strains under different temperature conditions (4°C, 20°C, 30°C and 40°C) and salinity (0.5 M, 1.0 M, 1.5 M, final concentration) on different carbon sources (CMC or xylan) was evaluated (Fig 4).

The fungi were able to grow on CMC in the absence of NaCl at 4°C and 30°C with optimal growth being observed at 20°C; TS2 (4.56 ± 0.13 mm/day), TS11 (7.11 ± 0.11 mm/day) and TS12 (5.85 ±0.15 mm/day) (Fig 4). Optimal growth rates were also observed at 20°C when the fungi were grown on CMC in the presence of 0.5 M and at 1.0 M NaCl in the case of both TS11 and TS12 suggesting that these fungi are psychrotolerant in nature, when growing on cellulose. The ability of these fungi to use CMC as a sole carbon source raises the possibility that in their natural habitats they may be able to use cellulose. Many members of the *Pseudoeurotiaceae* family to which *Pseudogymnoascus* belongs, grow saprotrophically on woody tissues and rotting vegetation and it is widely believed that they can degrade cellulosic substrates [102], while *Cadophora* is a natural wood-decay fungus [76,84,103].

With respect to fungal growth with xylan as a sole carbon source in the absence of NaCl, similar patterns were observed to those observed with CMC as a carbon source. Although no growth was detected in any of the strains at 40°C, growth was observed between 4°C and 30°C with optimal growth being observed at 20°C; TS2 (4.12 ± 0.14 mm/day), TS11 (7.43 ± 0.06 mm/day) and TS12 (7.90 ±0.08 mm/day) (Fig 4). Regarding the potential effect of salt on fungal growth, while no growth was observed at 1.5 M NaCl, the addition of 0.5 M NaCl to any of the fungi growing on either CMC or xylan as a sole carbon source did not significantly affect (*p*≤0.05) their growth at any temperature (Fig 4). However, increasing the salt concentration to 1.0 M had a noticeable negative effect (~2 to 5 mm/day and ~1.7 to 6 mm/day less than control treatment in all cases, on CMC and xylan respectively) on the daily growth rates of TS2, TS11 and TS12 (Fig 4). Interestingly, no growth was observed when TS11 and TS12 were grown at 40°C and 1.0 M NaCl, while limited growth was observed in TS2 (Fig 4), suggesting that while these fungi may exhibit halotolerance they are not thermotolerant under the conditions evaluated here. While strains TS2, TS11 and TS12 can be classified as halotolerant they are not halophilic given that their optimal growth parameters do not correspond to hypersaline conditions [104]. Given that these fungi were clearly capable of utilizing both CMC and xylan, we decided to study their cellulolytic and xylanolytic production potential.

### CMCase and xylanase activities

An initial Congo Red based plate assay with TS2, TS11 and TS12, indicated that they possessed both CMCase and xylanase activities (data not shown). Once the cellulolytic and xylanolytic potential of the strains was confirmed, a quantitative assay to monitor enzyme production in the three strains was performed. Maximum enzymatic activity for both enzymes was observed at day 9 of fermentation: CMCase activity (TS2: 8.11 ± 1.12 IU/mg protein, TS11: 3.89 ± 0.41 IU/mg protein, and TS12: 7.09 ± 0.66 IU/mg protein) and xylanase activity (TS2: 4.15 ± 0.61 IU/mg protein, TS11: 11.52 ± 1.28 IU/mg protein, and TS12: 9.2 ± 0.97 IU/mg protein) (specific activities measured at 30°C and pH 5). For this reason, the supernatants from day 9 were subsequently used to determine the optimum temperature and pH for xylanase and CMCase activity and to determine the halotolerance and thermostability of the enzymes.

### Effect of temperature and pH

**Fig 5:**
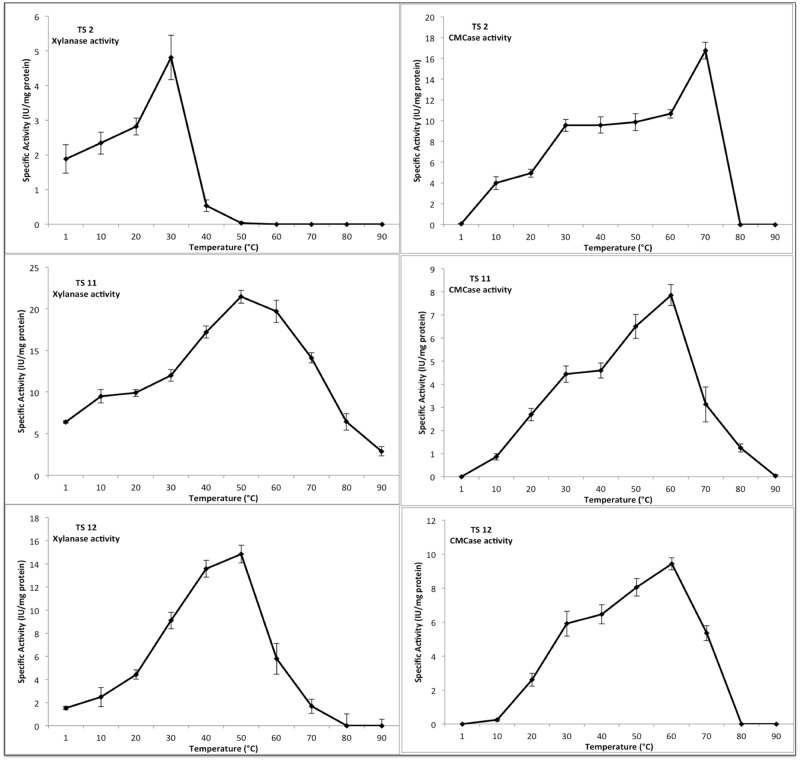
Optimal temperature for both CMCase and xylanase activities.

The optimal pH range for fungal CMCases and xylanases is generally in the pH 4 to 6 range [105–108]. Thus, we assessed the effect of temperature on CMCase and xylanase activity at pH 5. The optimum temperature for both CMCase and xylanase activity in each of the three fungi is shown in Fig 5. In the case of TS11 and TS12 optimal xylanase activity was observed at 50°C. In contrast, optimal xylanase activity in TS2 was observed at 30°C, with no activity being observed above 50°C. These activities are similar to those previously described for fungal xylanases. Optimal CMCase activity in TS11 and TS12 was observed at 60°C, while optimal activity in that TS2 was observed at 70°C. The CMCase activity observed in TS2 at 70°C was significantly higher (*p*≤0.05), than the activity observed in the other two fungal isolates (Fig 5). The optimal temperature observed for the CMCases of these three species is unusual in two ways: firstly, most of the fungal CMCases show a slightly lower optimal temperatures (50°C) [108] and secondly, it is worth noting that in TS2 the optimal CMCase temperature is markedly higher than the environment from which the fungus was originally isolated.

**Fig 6:**
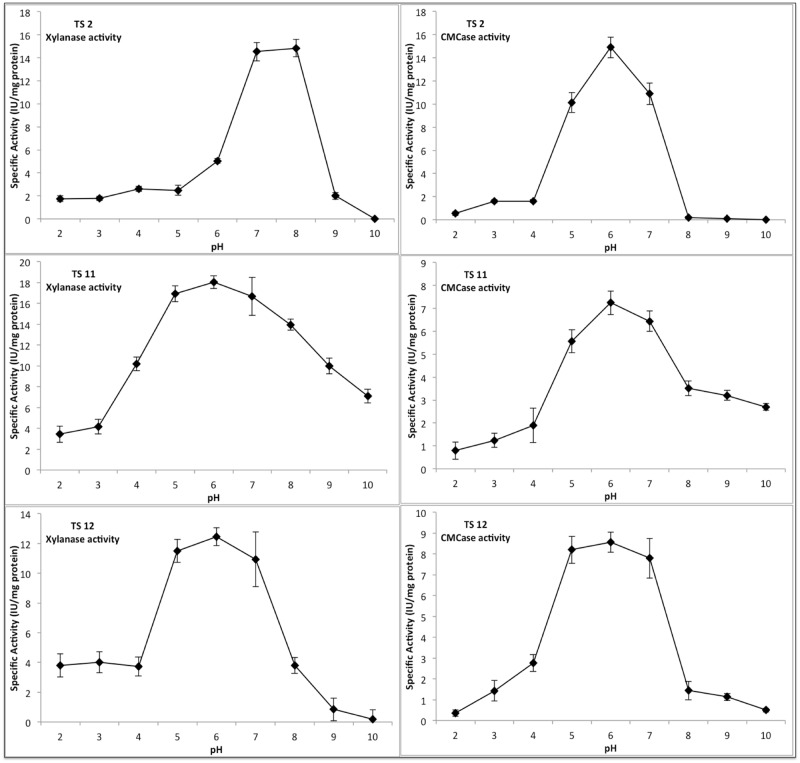
Optimal pH for both CMCase and xylanase activities.

The optimal pH for CMCase and xylanases activity in each fungal strain was then assessed (Fig 6). In general, the three strains showed a broad pH range for xylanases activity, with TS11 showing significant activity between 4 and 10. The optimal xylanases activity for TS11 and TS12 was 6, while interestingly TS2 showed an optimal pH at pH 8. The optimal activity for these xylanases is relatively high for this kind of fungal enzymes. Xylanases with optimal activity at higher pHs are of particular interest in the pulp and paper industry, since their use at higher pHs could reduce the levels of chlorine based chemicals required [109].

The optimal pH for CMCase in the three strains was between pH 5–6, but significant activity could still be observed at neutral pH and, in the case of TS11 even at pH 8. These pH values are also unusual for fungal CMCases, which are commonly optimally active in a more acidic range (pH 5).

### Thermostability of CMCases and xylanases

**Fig 7:**
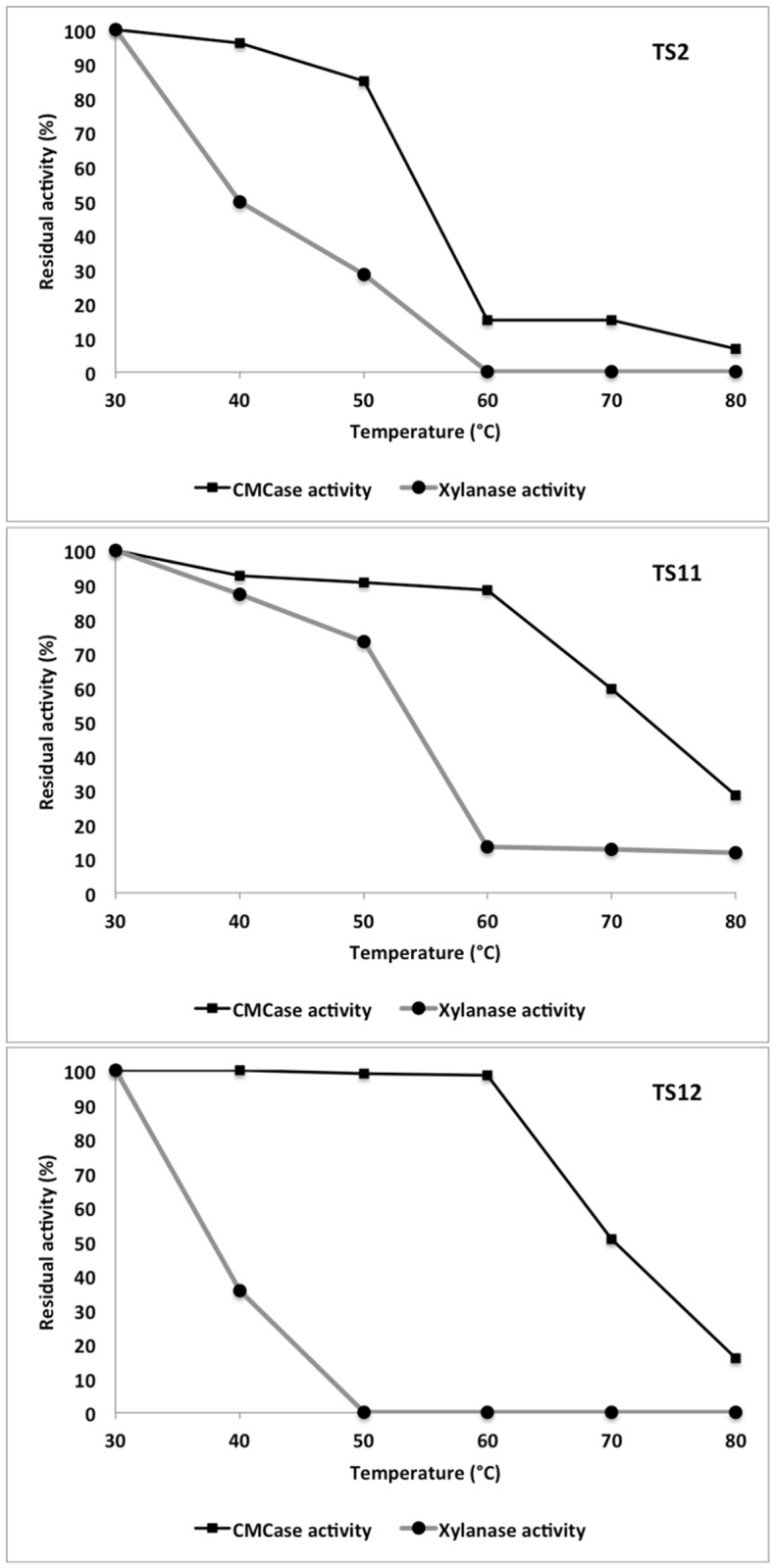
Thermostability of CMCase and xylanase activities.

TS11 produced the most thermostable CMCase activity retaining up to 28.38% of residual activity after incubating at 80°C for 1 h, while TS12 and TS2 retained lower levels of CMCase activity of 15.90% and 6.79% respectively (Fig 7).

In marked contrast while TS11 again produced the most thermostable xylanase activity retaining over 50.66% activity after 1 h at 70°C and up to 11.61% activity at 80°C, TS12 showed no xylanase activity after incubation of 50°C for 1 h, while TS2 displayed 28.45% residual activity at that temperature, but no activity at 60°C. Thus, TS11 produces the most thermostable enzymes (compared to TS2 and TS12) and may prove a suitable candidate for future genetic engineering based strategies to further improve this thermostability. The thermostability of the enzymes produced by these marine fungi are on average similar to other cellulases studied in the majority of fungal species, which is somewhat surprising given that they were isolated from a sponge at a depth of 751 m, where temperatures are around 3–8°C. However other extremophile fungi have previously been isolated from non-extreme habitats [66,110].

The level of CMCase thermostability reported in this study for TS2, TS11 and TS12 was better than those reported for *Bjerkandera adusta* and *Pycnoporus sanguineus*, even though the latter is a thermotolerant strain [70]. While the thermostability of the CMCases from these two basidiomycetes resulted in 94% of activity remaining when incubated below 50°C, when their supernatants were incubated at higher temperatures this activity was rapidly lost [70].

### Halotolerance of CMCase and xylanase activities

**Fig 8:**
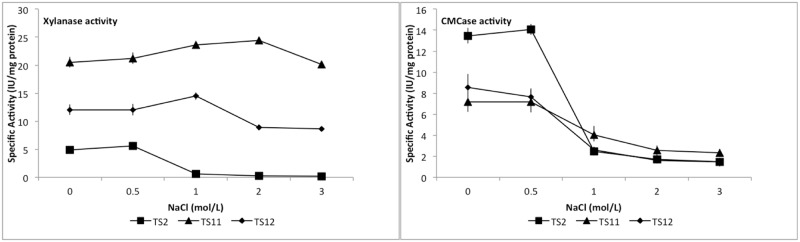
Halotolerance for both CMCase and xylanase activities.

The halotolerance of both CMCase and xylanase activities was evaluated by the addition of different salt (NaCl) concentrations to the reaction mixture (Fig 8). For CMCase, 0.5 M NaCl had no significant effect (*p*≤0.05) on the enzymatic activity when compared to no addition of salt. However, when the NaCl concentration was increased to 1 M a significant decrease in CMCase activity was observed for the three fungal strains (from 7.19 IU/mg protein and 7.66 IU/mg protein for TS11 and TS12 to 4.05 IU/mg protein and 2.60 IU/mg protein, respectively), with the most marked decrease occurring in TS2 (decrease from 14.06 IU/mg protein to 2.48 IU/mg protein). Further increases in NaCl concentrations to 2 M and 3 M showed similar effects (Fig 8). While there have been reports of bacterial cellulases that can withstand higher concentrations of salt than the cellulases reported here [111,112], nevertheless very little is known about halotolerant fungal cellulases [113]. During industrial processes such as denim dyeing for example, caustic soda, NaCl and other chemicals are used, so halotolerant cellulases which would remain active under those processing conditions would be useful; These fungal cellulases would be particularly attractive for this process given that the range of salt concentrations used for denim dyeing is around 0.14 M NaCl, which is much lower than the levels at which these fungal cellulases are optimally active.

In the case of xylanolytic activity, no statistical difference (*p*≤0.05) in relation to enzymatic activity in any of the fungal strains was observed at either 0 M or 0.5 M NaCl concentrations. However, increases in xylanolytic activity in TS11 and TS12 from 21.23 IU/mg protein to 26.63 IU/mg protein and from 12.07 IU/mg protein to 14.50 IU/mg protein respectively, were observed in the presence of 1 M NaCl. Conversely, xylanolytic activity in TS2 decreased from 5.63 IU/mg protein to almost non-detectable levels, when the salt concentration was above 1 M. Moreover, xylanolytic activity in TS11 continued to be high when the NaCl concentration increased to 2 M, and decreased thereafter at 3 M; which was in marked contrast to the effect exhibited in TS2 and TS12 (Fig 8).

The presence of cellulase activity in these three fungal genera has been reported previously [103,114]. *Cadophora malorum* isolated from an expedition hut on Ross Island, Antarctica has been reported to exhibit a strong cellulase activity (>100 IU/mg protein) at psychrophilic temperatures (4 and 15°C) [114]. *Emericellopsis* spp. have also been shown to produce cellulase activity on solid media [115], while cellulases are thought to play a role in the infection of bats by *Pseudogymnoascus* spp. [91]. However, this is the first report of CMCase activity and indeed of xylanase activity in these three fungi isolated from a marine sponge.

### Solid state fermentation

Although TS2, TS11 and TS12 are marine-derived fungi living in an aqueous environment, fermentations under water-limited conditions on wheat straw and corn stover were performed. Large quantities of both residues are annually produced and stored, and they are under-exploited [116]. Thus, fungi with the ability to colonize them are attractive for the recycling of these agro-based residues and its downstream applications.

Due to the potential utility of these three fungi in biomass conversion we assessed the production of CMCases, xylanase and peroxidase/phenol oxidase activities in cultures grown on natural lignocellulosic materials (wheat straw and corn stover). The latter enzymes are involved in lignin degradation, thus making cellulose and hemicellulose readily available to cellulases and xylanases. The production of these tripartite activities would allow fungi to colonize lignocellulosic wastes such as wheat straw and corn stover. The three fungi colonized both substrates, with slightly higher overall enzyme titres being produced on corn stover than on wheat straw (Table 1). Higher levels of xylanase activity than CMCase activity were observed on both substrates. Xylanase activity was measured in all three fungi on wheat straw, with the highest levels observed in TS12 (2.33 ± 0.20 IU/mg protein). CMCase activity levels were the highest levels again being observed in TS12 (0.76 ± 0.11 IU/mg protein) on wheat straw. Avicelase activity was highest for TS11 in both substrates, but significant for the other strains in corn stover as well as in wheat straw.

**Table 1:** Total proteins and enzymatic activities (expressed in IU/mg protein) in supernatants collected from the SSF.

Strain	SSF on wheat straw					SSF on corn stover				
	Prot	Activities from supernatants				Prot	Activities from supernatants			
		CMCase	Xyl	Pox	Avi		CMCase	Xyl	Pox	Avi
**TS2**	0.86 ± 0.05	0.38 ± 0.10	1.87 ± 0.21	127.82 ± 3.79	0.91 ± 0.11	2.53 ± 0.31	0.47 ± 0.09	0.75 ± 0.11	114.17 ± 3.77	1.03 ± 0.17
**TS11**	1.23 ± 0.19	0.67 ± 0.13	1.83 ± 0.37	99.84 ± 3.88	1.20 ± 0.26	2.38 ± 0.30	0.42 ± 0.05	1.16 ± 0.14	104.95 ± 4.09	1.36 ± 0.25
**TS12**	0.99 ± 0.10	0.76 ± 0.11	2.33 ± 0.20	118.37 ± 4.11	0.88 ± 0.32	1.40 ± 0.19	0.43 ± 0.07	0.80 ± 0.08	114.25 ± 3.04	0.72 ± 0.10

Prot (Total protein), Xyl (xylanase) and Pox (phenol oxidase). Avi (Avicelase)

Regarding peroxidase/phenol oxidase activity (Pox), the method employed allowed the assessment of both peroxidase and phenol oxidase enzymatic activities [68]; Pox activity was observed in all cultures on both substrates with the higher overall levels again being observed in wheat straw, and the highest Pox levels being observed in TS2 (127.82 ± 3.79 IU/mg protein). The fact that we found Pox activity in these fungi suggests they have the capacity to degrade lignin, a major component in plant biomass wastes. This is to the best of our knowledge, the first time that peroxidase/phenol oxidase activity has been reported in these three fungal genera. This together with the fact that TS2, TS11 and TS12 displayed CMCase and xylanase activities on these agricultural waste materials, suggests that they may be good candidates for further evaluation in fungal mediated lignocellulosic biomass conversion strategies; particularly for biorefinery related biotechnological applications.

### Zymograms for CMCases and xylanases

**Fig 9:**
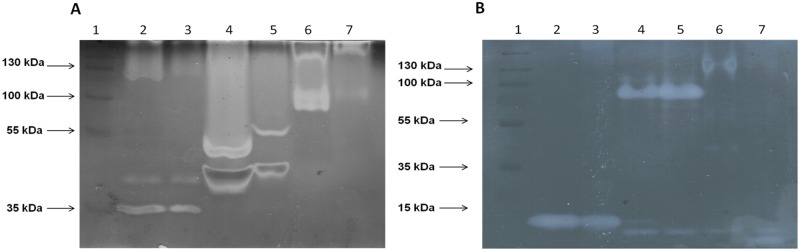
Zymogram for detection of CMCases and xylanases in different natural substrates. (A) CMCase activity bands, 1) Molecular weight marker, 2) Strain TS2 wheat straw supernatant, 3) Strain TS2 corn stover supernatant, 4) Strain TS11 wheat straw supernatant, 5) Strain TS11 corn stover supernatant, 6) Strain TS12 wheat straw supernatant, 7) Strain TS12 corn stover supernatant. (B) Xylanase activity bands, 1) Molecular weight marker, 2) Strain TS2 wheat straw supernatant, 3) Strain TS2 corn stover supernatant, 4) Strain TS11 wheat straw supernatant, 5) Strain TS11 corn stover supernatant, 6) Strain TS12 wheat straw supernatant, 7) Strain TS12 corn stover supernatant.

Although a number of enzyme activities (CMCases, xylanases, peroxidase/phenol oxidase) were detected in the supernatants obtained from the fungi (TS2, TS11 and TS12) grown under SSF, our primary focus was to further characterize the CMCase and xylanase activities. To this end we used zymographic methods to monitor the production of CMCase and xylanase isoforms, (Fig 9) to corroborate the activities reported in Table 1.

Three bands of approximately 35 kDa, 40 kDa and 120 kDa corresponding to CMCase activity were observed in TS2 grown in both wheat straw and corn stover (lanes 2 and 3, Fig 9A). In contrast, two major bands were observed when TS11 was grown on each substrate: one band of 40 kDa for both substrates, and another of 50 kDa when the strain was grown on wheat straw and 55 kDa corresponding to CMCase activity, when TS11 was grown on corn stover (lanes 4 and 5, Fig 9A). Three bands of approximately 80 kDa, 100 kDa and 130 kDa were observed when TS12 was grown on wheat straw, while two bands (130 kDa and 100 kDa) were visualized when TS12 was grown on corn stover (lanes 6 and 7, Fig 9A). Thus it is clear that CMCase production in TS11 and TS12 is affected by the substrate. This has previously been reported in both *Trichoderma* sp. and in *Bjerkandera adusta* where different cellulolytic profiles are produced when the fungi are grown on various natural substrates [105,117].

With respect to xylanase activity one predominant band of approximately 10 kDa was observed when TS2 was grown on both substrates (lanes 2 and 3, Fig 9B). A major band of around 100 kDa was observed when TS11 was grown on both substrates, with other smaller bands also being observed (lanes 4 and 5, Fig 9B). A specific band (approximately 10 kDa) of xylanase can also be observed in lane 4 (Fig 9B), indicating substrate dependent xylanolytic production. Low molecular weight xylanases were mainly produced by TS12 (lanes 6 and 7), Fig 9B. TS12 also produced different xylanase isoforms when it was grown on both substrates. This has also previously been reported for the white rot fungus *Pycnoporus sanguineus* [105]. The low (≅12 kDa) and medium (≅35 kDa) molecular weight CMCases and xylanases, which are produced by these three fungi (Fig 9B), are particularly attractive from the perspective of biotechnological processes for biomass conversion. Because of the tightly polymer packaging present in the plant cell wall it is necessary to find new low molecular weight enzymes to increase the efficiency of the biomass degradation [118]. In addition low molecular weight enzymes frequently show interesting biochemical properties such as thermotolerance and high stability [119], and are typically good candidates for overexpression and protein engineering based approaches to improve their physical and biochemical properties.

### Saccharification of cotton fibres with enzyme crude extracts

**Fig 10:**
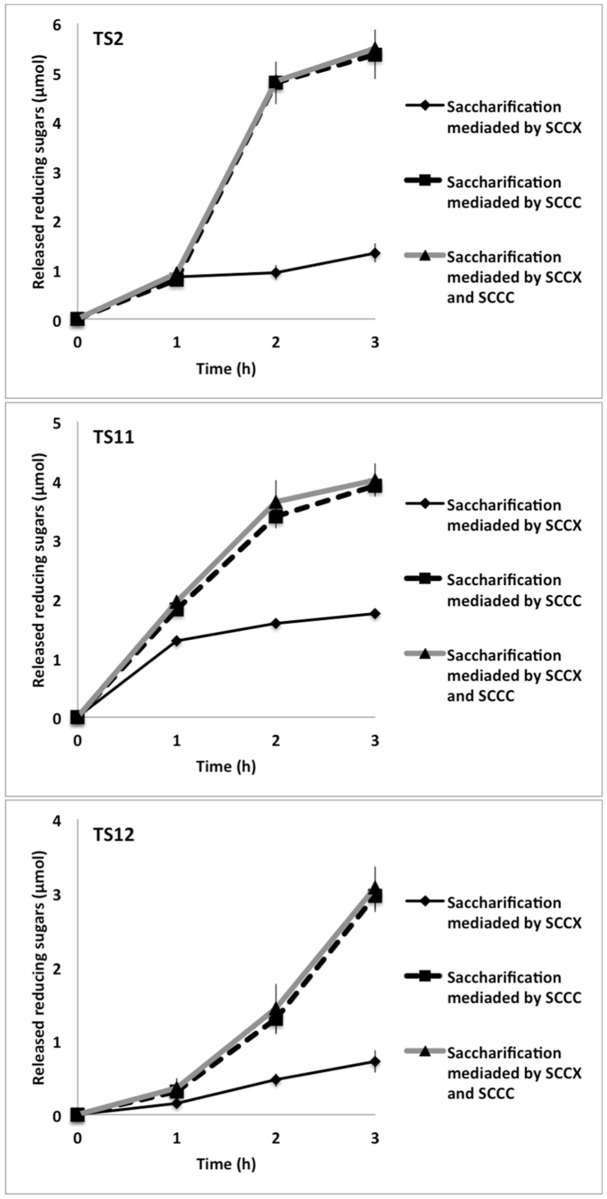
Saccharification of cotton fibres. SCCX: Supernatants Collected from Cultures grown with Xylan. SCCC: Supernatants Collected from Cultures grown with CMC.

Given that the three fungi exhibited cellulolytic and xylanolytic activities we evaluated their saccharification potential with mercerized cotton fibres (Fig 10). Supernatants of TS11 grown with xylan as carbon source (in which both cellulases and xylanases can be produced, see below) exhibited the best saccharification of the mercerized cotton fibres, with 1.75 μmol of reducing sugars being released after 3 h incubation; which was 2.4 and 2.0 times more reducing sugars released than TS12 and TS2 supernatants, respectively (Fig 10). Cellulase secretion or the presence of xylanases with cellulase activity in the supernatants is consistent with the fermentable sugars we observed when cotton fibres were incubated with supernatants of fungal cultures grown on xylan. Another possibility is that a small amount of xyloglucan is still present in the cotton fibres, so part of the reducing sugars observed may come from this substrate. However, it is worth noting that under these conditions the lowest amount of liberated reducing sugars was observed, suggesting either lower cellulase production or a lower content of xyloglucan with respect to cellulose in the cotton fibres. When CMC was used as a carbon source, TS2 exhibited the best potential, releasing 5.37 μmol of reducing sugars after 3 h incubation; which is 1.8 and 1.4 times more than TS12 and TS11, respectively (Fig 10). The fibre saccharification using supernatants from all fungi grown in the presence of CMC was higher than those obtained when cotton was incubated with supernatants collected of fungal cultures grown with xylan as the carbon source. These results are consistent with the chemical composition of the fibres. No increase in saccharification of the cotton fibres was observed when supernatants from both CMC and xylan cultures were mixed together. The degree of saccharification obtained with supernatants from the CMC cultured fungi is comparable to those previously obtained by our group using commercial cellulase preparations [120]; (4.25 micromoles released from CMC using commercial cocktails compared to 5–6 micromoles released from cotton fibres obtained here), demonstrating that these fungi clearly display interesting biotechnological potential. Incubations after 3 h do not show an increase of sugar liberation in any case (Fig 10), suggesting that all the “degradable” cellulose was hydrolyzed.

Finally, Avicel was used to confirm the presence of cellulases in supernatants from fungal-liquid cultures. These results show that TS2, TS11 and TS12 strains produced cellulases under submerged conditions. Cellulases were detected in fungal cultures with CMC as a sole carbon source, while supernatants derived from fungi grown on xylan as a sole carbon source did not show cellulases activities. TS2 showed highest levels of activity (1.39 ± 0.37 IU/mg protein), while 1.20 ± 0.22 IU/mg protein and 0.97 ± 0.17 IU/mg protein were detected in TS11 and TS12, respectively.

In summary, we present here the isolation of three marine fungal species associated with the deep-sea sponge *Stelletta normani* and characterisation of their CMCase, xylanases, avicelase and Pox activities. Interestingly, CMCase activities from the three fungi were thermostable *in vitro*, which is surprising given the habitat from which the strains were collected (with average temperatures of 3–8°C). However, other selective “forces” such as high pressure (at 750 m below sea level) or high salinity may have helped select for these features in these proteins. CMCase activity from each of the strains was shown to be composed of two to three protein bands in zymograms and in the case of TS11, was differentially expressed depending on the substrate used for growth. In the case of xylanase activity, zymograms revealed that one or two protein bands contributed to this activity. Xylanase activity from strains TS2 and TS11 was observed in the presence of high NaCl concentrations, while CMCase activity from the three isolates was also observed in the presence of at least 0.5 M NaCl. The halotolerance of these enzymes is perhaps not surprising given the marine origin of the isolates. Saccharification of cotton fibres (a recalcitrant substrate) released an amount of sugars comparable to those obtained by commercial cellulases cocktails [120].

Thus while it is clear that the precise role of fungi associated with marine sponges has yet to be fully elucidated, nonetheless these fungi appear to be a good source of potential novel biocatalysts with interesting biochemical and physical properties. It is clear that fungal communities associated with deep-sea sponges represent an extraordinary resource with respect to biocatalytic potential, and in particular as demonstrated here, with respect to lignocellulose degradation which is continuing to attract much interest due to the on-going need for improved biomass conversion strategies.

## Supporting information

S1 Fig TS2 18S rDNA gene phylogeny. Sequence deposited under accession number KR336667. Phylogeny was conducted using BioNJ with K2P as substitution model. Bootstrap values are indicated with corresponding nodes. Bar indicates the nucleotide substitution per site. *Ustilago maydis* was used as outgroup.
(TIF)

S2 Fig TS11 18S rDNA gene phylogeny. Sequence deposited under accession number KR336668. Phylogeny was conducted using BioNJ with K2P as substitution model. Bootstrap values are indicated with corresponding nodes. Bar indicates the nucleotide substitution per site. *Ustilago maydis* was used as outgroup.
(TIF)

S3 Fig TS12 18S rDNA gene phylogeny. Sequence deposited under accession number KR336669. Phylogeny was conducted using BioNJ with K2P as substitution model. Bootstrap values are indicated with corresponding nodes. Bar indicates the nucleotide substitution per site. *Ustilago maydis* was used as outgroup.
(TIF)

S1 Table Ribosomal sequences used to produce tree reconstructions. (DOCX)

S2 Table First hits retrieved from the blastn according with ITS1, ITS2, D1-D2 and 18S rRNA sequences for each new fungal isolate. (DOCX)
