# Supplementary figures and images for: Characterization of lignocellulolytic activities from fungi isolated from the deep-sea sponge Stelletta normani
Source: PLoS One. 2017 Mar 24;12(3):e0173750. doi: 10.1371/journal.pone.0173750 (PMC5365110; doi:10.1371/journal.pone.0173750)

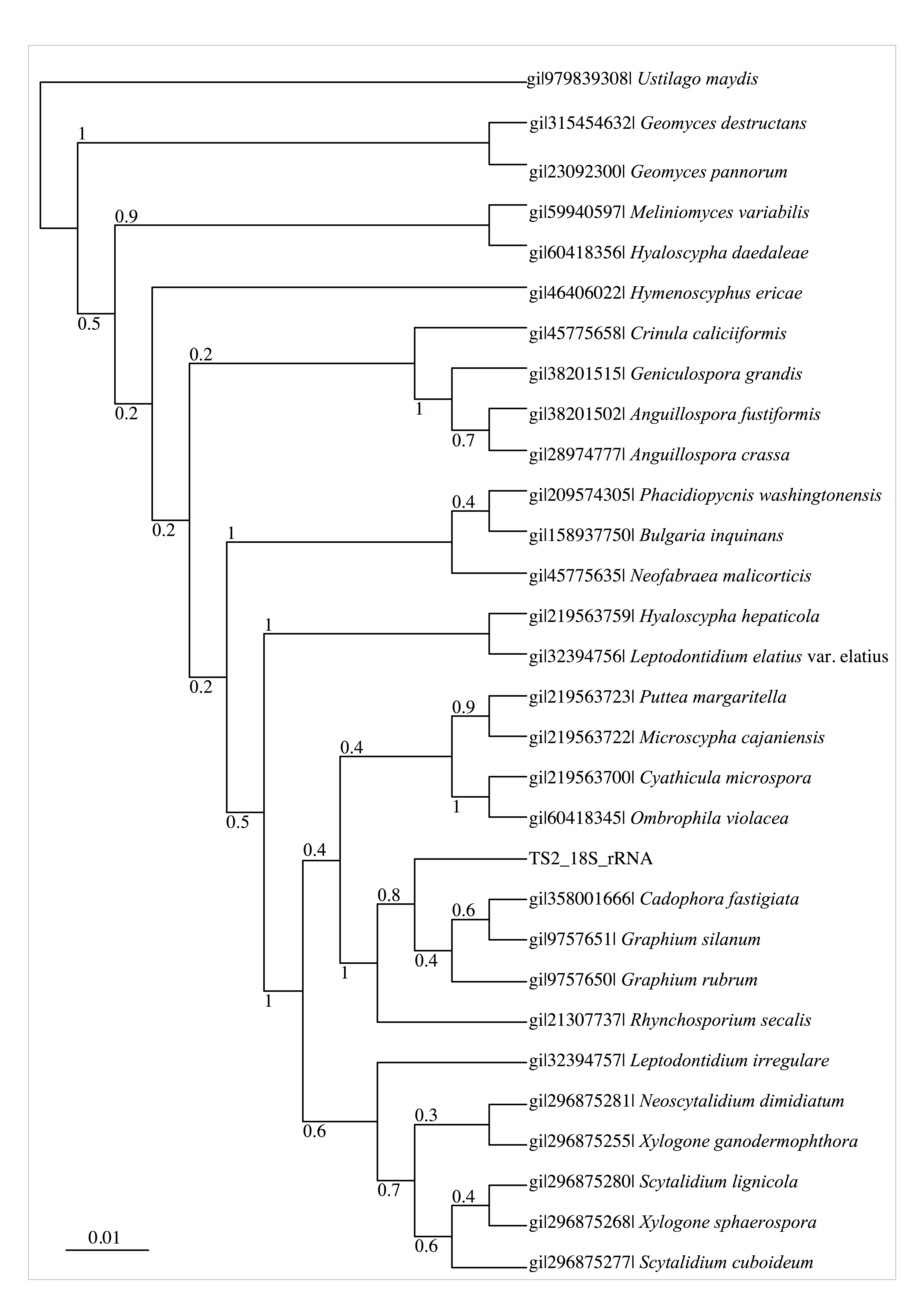

Supplement: S1 Fig — Sequence deposited under accession number KR336667. Phylogeny was conducted using BioNJ with K2P as substitution model. Bootstrap values are indicated with corresponding nodes. Bar indicates the nucleotide substitution per site. Ustilago maydis was used as outgroup. (TIF) [file pone.0173750.s001.tif]

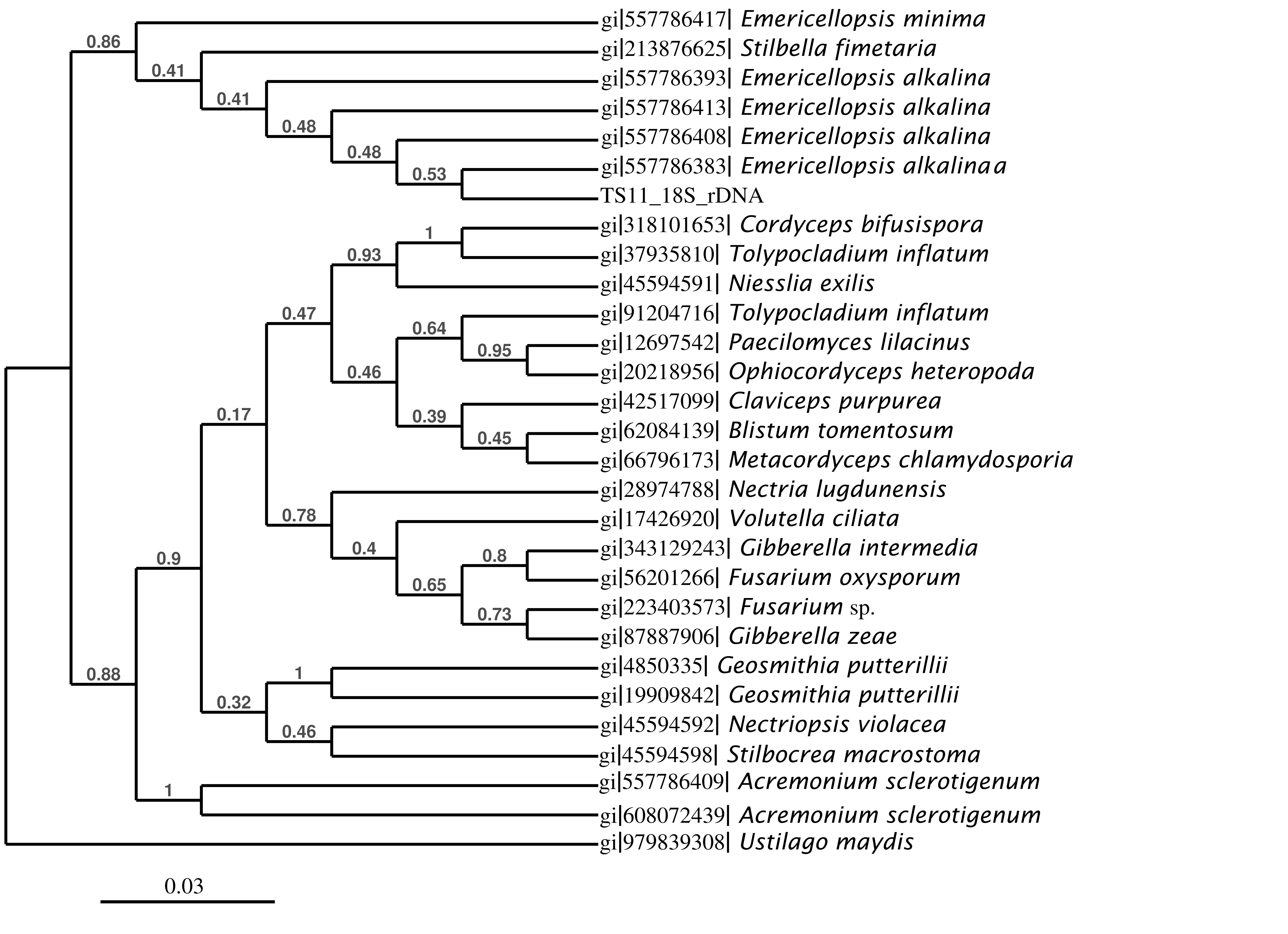

Supplement: S2 Fig — Sequence deposited under accession number KR336668. Phylogeny was conducted using BioNJ with K2P as substitution model. Bootstrap values are indicated with corresponding nodes. Bar indicates the nucleotide substitution per site. Ustilago maydis was used as outgroup. (TIF) [file pone.0173750.s002.tif]

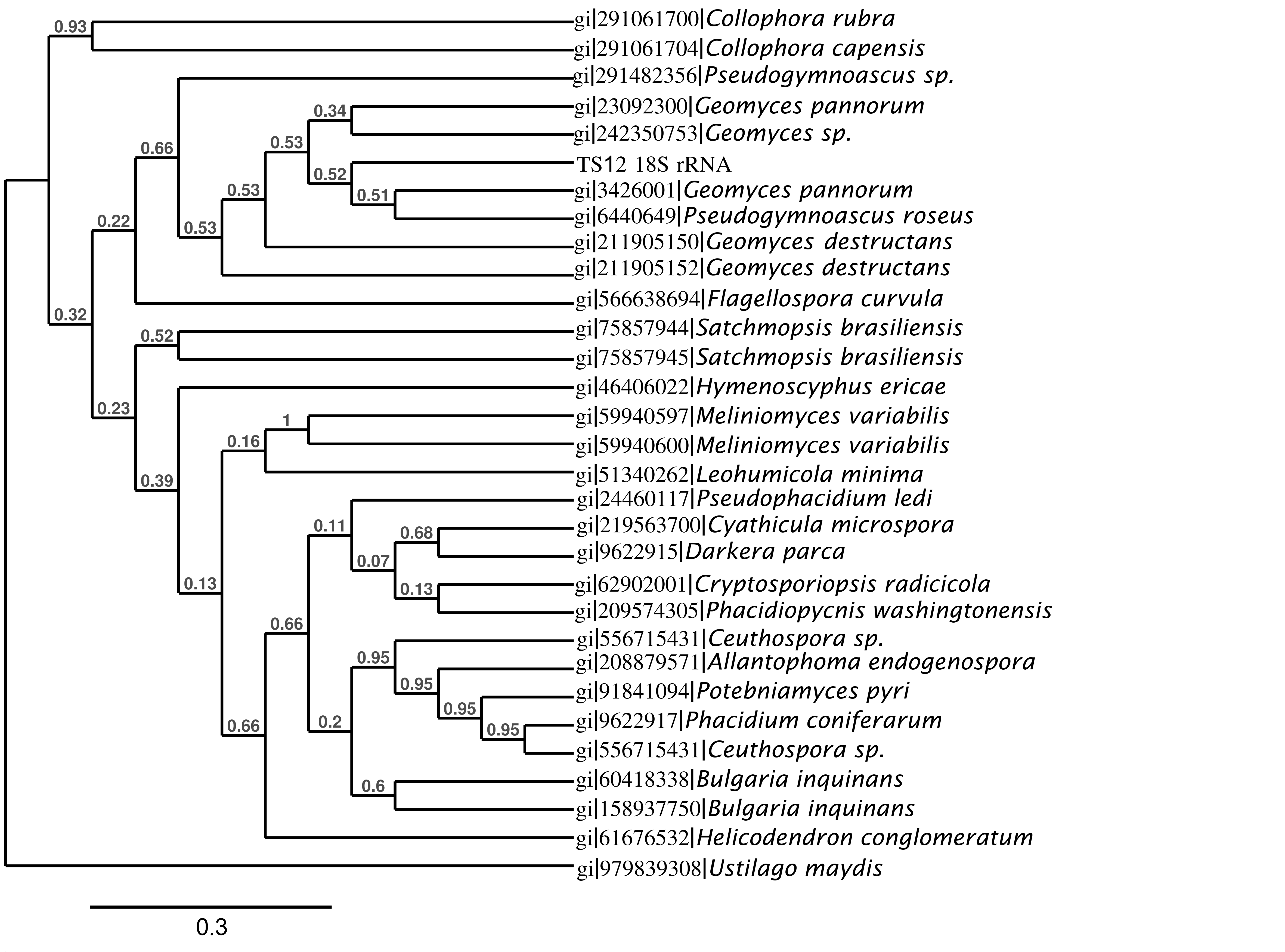

Supplement: S3 Fig — Sequence deposited under accession number KR336669. Phylogeny was conducted using BioNJ with K2P as substitution model. Bootstrap values are indicated with corresponding nodes. Bar indicates the nucleotide substitution per site. Ustilago maydis was used as outgroup. (TIF) [file pone.0173750.s003.tif]
