# Supplementary material for: Characterization of lignocellulolytic activities from fungi isolated from the deep-sea sponge Stelletta normani
Source: PLoS One. 2017 Mar 24;12(3):e0173750. doi: 10.1371/journal.pone.0173750 (PMC5365110; doi:10.1371/journal.pone.0173750)
Supplement: S1 Table — (DOCX) [file pone.0173750.s004.docx]

S1 Table 1. Ribosomal sequences used to produce tree reconstructions.

| **Organism** | **Accession number** | | |
| --- | --- | --- | --- |
|  | **ITS1** | **ITS2** | **D1-D2** |
| *Pseudogymnoascus roseus* | gi\|75265678\| | gi\|75265678\| | gi\|177221573\| |
| *Pseudogymnoascus pannorum* | gi\|675895373\| | gi\|675895373\| | gi\|17221586\| |
| *Pseudogymnoascus destructans* | gi\|566601019\| | gi\|566601019\| | gi\|441037390\| |
| *Pseudeurotium ovale* | gi\|807045072\| | gi\|807045072\| | gi\|807045073\| |
| *Oidiodendron tenuissimum* | gi\|54126047\| | gi\|54126047\| | gi\|17221589\| |
| *Oidiodendron maius* | gi\|54126050\| | gi\|54126050\| | gi\|367057829\| |
| *Myxotrichum deflexum* | gi\|3687772\| | gi\|3687772\| | gi\|17221572\| |
| *Leptodontium elatius* | gi\|32394761\| | gi\|32394761\| | gi\|51101427\| |
| *Geomyces pannorum* a | gi\|75265671\| | gi\|75265671\| | gi\|294959352\| |
| *Geomyces pannorum* b | gi\|75265669\| | gi\|75265669\| | gi\|294959350\| |
| *Geomyces auratus* | gi\|541136164\| | gi\|541136164\| | gi\|541136152\| |
| *Ciliciopodium hyalinum* | gi\|808213319\| | gi\|808213319\| | gi\|808212609\| |
| *Ciliciopodium brevipes* | gi\|808213318\| | gi\|808213318\| | gi\|808212608\| |
| *Chrysosporium zonatum* | gi\|68445665\| | gi\|68445665\| | gi\|563257374\| |
| *Chrysosporium tropicum* | gi\|807045329\| | gi\|807045329\| | gi\|563257372\| |
| *Acremonium dichromosporum* | gi\|452056266\| | gi\|452056266\| | - |
| *Acremonium exoviarum* | gi\|189307044\| | gi\|189307044\| | - |
| *Acremonium fuci* | gi\|49035532\| | gi\|49035532\| | - |
| *Aspergi\|llus caesiellus* | gi\|34809345\| | gi\|34809345\| | - |
| *Aspergi\|llus insuetus* | gi\|158535918\| | gi\|158535918\| | - |
| *Aspergi\|llus kevei* | gi\|158535893\| | gi\|158535893\| | - |
| *Aspergi\|llus niveus* | gi\|34809333\| | gi\|34809333\| | - |
| *Aspergi\|llus occultus* | gi\|665387848\| | gi\|665387848\| | - |
| *Aspergi\|llus pulvericola* | gi\|665387845\| | gi\|665387845\| | - |
| *Aspergi\|llus tabacinus* | gi\|158535939\| | gi\|158535939\| | - |
| *Aspergi\|llus westlandensis* | gi\|665387840\| | gi\|665387840\| | - |
| *Emericella cleistominuta* | gi\|119359832\| | gi\|119359832\| | - |
| *Emericella discophora* | gi\|184161560\| | gi\|184161560\| | - |
| *Emericella montenegro* | gi\|119359813\| | gi\|119359813\| | - |
| *Emericella stella-maris* | gi\|184161565\| | gi\|184161565\| | - |
| *Emericellopsis salmosynnemata* | gi\|49035546\| | gi\|49035546\| | - |
| *Emericellopsis synnematicola* | gi\|49035545\| | gi\|49035545\| | - |
| *Emericellopsis pallida* | gi\|49035547\| | gi\|49035547\| | - |
| *Emericellopsis maritima* | gi\|671756968\| | gi\|671756968\| | - |
| *Emericellopsis minima* | gi\|49035549\| | gi\|49035549\| | - |
| *Emericellopsis mirabilis* | gi\|49035536\| | gi\|49035536\| | - |
| *Emericellopsis pusilla* | gi\|197313487\| | gi\|197313487\| | - |
| *Emericellopsis glabra* | gi\|332113245\| | gi\|332113245\| | - |
| *Emericellopsis robusta* | gi\|49035544\| | gi\|49035544\| | - |
| *Eurotium echinulatum* | gi\|158535206\| | gi\|15853520\|6 | - |
| *Eurotium umbrosum* | gi\|158535200\| | gi\|15853520\| | - |
| *Eurotium tonophilum* | gi\|158535226\| | gi\|158535226\| | - |
| *Eurotium heterocaryoticum* | gi\|158138945\| | gi\|158138945\| | - |
| *Eurotium leucocarpum* | gi\|158535232\| | gi\|158535232\| | - |
| *Eurotium halophilicum* | gi\|158535233\| | gi\|158535233\| | - |
| *Fusarium oxysporum* | gi\|908842234\| | gi\|908842234\| | - |
| *Nectria pseudotrichia* | gi\|386684329\| | gi\|386684329\| | - |
| *Stanjemonium ochroroseum* | gi\|49035552\| | gi\|49035552\| | - |
| *Stanjemonium grisellum* | gi\|49035551_1\| | gi\|49035551_1\| | - |
| *Stibella fimetaria* | gi\|298106289\| | gi\|298106289\| | - |
| *Trichoderma reesei* | gi\|914330185\| | gi\|914330185\| | - |
| *Phialocephala trigonospora* | gi\|184127833\| | gi\|184127833\| | gi\|452085143\| |
| *Phialocephala xalapensis* | gi\|20136205\| | gi\|20136205\| | gi\|11527232\| |
| *Phialocephala humicola* | gi\|452085164\| | gi\|452085164\| | gi\|452085142\| |
| *Phialocephala virens* | gi\|20136206\| | gi\|20136206\| | gi\|452085144\| |
| *Phialocephala fortinni* | gi\|19347633\| | gi\|19347633\| | gi\|204343778\| |
| *Graphium jumulu* | gi\|828364321\| | gi\|828364321\| | gi\|828364366\| |
| *Graphium scolytodis* | gi\|115501966\| | gi\|115501966\| | gi\|115501965\| |
| *Mollisia incrustata* | gi\|290783862\| | gi\|290783862\| | gi\|290783862\| |
| *Leptodontidium elatius* | gi\|32394761\| | gi\|32394761\| | gi\|51101427\| |
| *Cadophora melini* | gi\|89257851\| | gi\|89257851\| | gi\|29825795\| |
| *Cadophora fastigata* | gi\|30144896\| | gi\|30144896\| | gi\|307548433\| |
| *Cadophora luteo-olivacea* | gi\|262385611\| | gi\|262385611\| | gi\|29825791\| |
| *Cadophora malorum* | gi\|262385614\| | gi\|262385614\| | gi\|29825796\| |
| *Ustilago maydis** | gi\|194371712\| | gi\|194371712\| | gi\|546471615\| |

*Ribosomal sequences from *U. maydis* were used as outgroup in all phylogenies.
