# Supplementary material for: Characterization of lignocellulolytic activities from fungi isolated from the deep-sea sponge Stelletta normani
Source: PLoS One. 2017 Mar 24;12(3):e0173750. doi: 10.1371/journal.pone.0173750 (PMC5365110; doi:10.1371/journal.pone.0173750)
Supplement: S2 Table — (DOCX) [file pone.0173750.s005.docx]

S1 Table 2. First hits retrieved from the blastn according with ITS1, ITS2, D1-D2 and 18S rRNA sequence for each new fungal isolate.

| **Strain** | **Mol. marker** | **Nucleotide blast (first hits)** | **e-value** | **id.** | **cov.** |
| --- | --- | --- | --- | --- | --- |
| **TS2** | ITS1 | *Cadaphora malorum* TS_06_017 | 1e-131 | 99% | 100% |
|  | ITS2 | *Cadaphora* sp. P1381 | 2e-139 | 100% | 100% |
|  | D1-D2 | Fungal endophyte voucher* | 0.0 | 99% | 100% |
|  | 18S rRNA | Helotiales sp. MF580**^◆^** | 0.0 | 100% | 100% |
| **TS11^◼^** | ITS1 | *Emericellopsis pallida* DM12 | 3e-102 | 100% | 100% |
|  | ITS2 | *Acremonium zonatum* JCKQF3**^✪^** | 1e-146 | 100% | 100% |
|  | 18S rRNA | *Acremonium* sp. Y39-2**^✪^** | 0.0 | 100% | 100% |
| **TS12** | ITS1 | Fungal sp. MKOTU119**^⌃^** | 9e-113 | 100% | 100% |
|  | ITS2 | *Geomyces* sp. S7-Z-2-7 | 1e-137 | 99% | 100% |
|  | D1-D2 | *Geomyces pannorum* 38249-10 | 0.0 | 100% | 99% |
|  | 18S rRNA | Fungal sp. ZJ59**^★^** | 0.0 | 100% | 100% |

Mol. Marker, molecular markers; id., identity; cov., coverage.

*The first ten hits retrieved from the blastn describe *Phialocephala* sp. and *Cadophora* sp. strains. In all cases, hits show: 99-100% of query cover, 0.0 of E value and 99% of identity.

**^◆^**Helotiales is not a fungal genus. The second and third hits retrieved from the blastn belong to Cadophora fastigiata and Cadophora luteo-olivacea. The three hits show: 100% of query cover, 0.0 of E value and 100% of identity.

**^◼^**D1-D2 ribosomal region did not amplify from genomic DNA isolated from TS11 strain.

**^✪^**The first ten hits retrieved from the blastn describe *Acremonium* sp. and *Emericellopsis* sp. strains. In all cases, hit show: 99-100% of query cover, e-145 or 0.0 of E value and 99-100% of identity.

**^⌃^**The first ten hits retrieved from the blastn describe *Pseudogymnoascus* sp. strains. In all cases, hit show: 100% of query cover, 9e-113 of E value and 100% of identity.

**^★^**The first ten hits retrieved from the blastn describe *Geomyces* sp. and *Pseudogymnoascus* sp. strains. In all cases, hit show: 100% of query cover, 0.0 of E value and 100% of identity.
